# Critcomms: a national cross-sectional questionnaire based study to investigate prehospital handover practices between ambulance clinicians and specialist prehospital teams in Scotland

**DOI:** 10.1186/s13049-018-0512-3

**Published:** 2018-06-01

**Authors:** David Fitzpatrick, Michael McKenna, Edward A. S. Duncan, Colville Laird, Richard Lyon, Alasdair Corfield

**Affiliations:** 1 Faculty of Health Sciences and Sport, University of Stirling, FK9 4LA Stirling, Scotland; 2 Scottish Ambulance Service, Glebe Cottage, Strath, Gairloch, Ross-shire IV212BT Scotland; 3 Nursing, Midwifery & Allied Health Professions Research Unit, University of Stirling, FK9 4NF Scion House, Scotland, UK; 4 Basics Scotland, Aberuthven Enterpise Park, Sandpiper House, Aberuthven, Auchterarder Scotland; 5 Pre-Hospital Emergency Care, School of Health Sciences, University of Surrey, Guildford, UK; 6 Emergency Medical Retrieval Service, School of Medicine, Dentistry and Nursing, University of Glasgow, Wolfson Medical School Building, G12 8QQ Glasgow, Scotland

**Keywords:** Handover, Paramedic, Prehospital, Critical care teams, HEMS, Safety, Quality, Mnemonics

## Abstract

**Background:**

Poor communication during patient handover is recognised internationally as a root cause of a significant proportion of preventable deaths. Improving the accuracy and quality of handover may reduce associated mortality and morbidity. Although the practice of handover between Ambulance and Emergency Department clinicians has received some attention over recent years there is little evidence to support handover best practice within the prehospital domain. Further research is therefore urgently required to understand the most appropriate way to deliver clinical information exchange in the pre-hospital environment. We aimed to investigate current clinical information exchange practices, perceived challenges and the preferred handover mnemonic for use during transfer of high acuity patients between ambulance clinicians and specialist prehospital teams.

**Methods:**

A national, cross-sectional questionnaire study. Participants were road based ambulance clinicians (RBAC) or active members of specialist prehospital teams (SPHT) based in Scotland.

**Results:**

Over a three month study period there were 247 prehospital incidents involving specialist teams. One hundred ninety individuals completed the questionnaire; 61% [*n* = 116] RBAC and 39% [*n* = 74] SPHT. Median length of prehospital experience was 10 years (IQR 5–18). Overall current prehospital handover practices were perceived as being effective (Mdn 4.00; IQR 3–4 [1 = very ineffective - 5 = very effective]) although SPHT clinicians rated handover effectiveness slightly lower than RBAC’s (Mdn 3.00 vs 4.00, U = 1842.5, *p* = .03). ‘ATMIST’ (Age, Time of onset, Medical complaint/injury, Investigation, Signs and Treatment) was deemed the mnemonic of choice. The clinical variables perceived as essential for handover are not explicitly identified within the SBAR mnemonic. The most frequently reported method of recording and transferring information during handover was via memory (*n* = 112 and *n* = 120 respectively) and ‘*interruptions*’ were perceived as the most significant barrier to effective handover.

**Conclusion:**

While, overall, current prehospital handover practice is perceived as effective this study has identified a number of areas for improvement. These include the development of a shared mental model through system standardisation, innovations to support information recording and delivery, and the clear identification at incidents of a handover lead. Mnemonics must be carefully selected to ensure they explicitly contain the perceived essential clinical variables required for prehospital handover; the mnemonic ATMIST meets these requirements. New theoretically informed, evidence-based interventions, must be developed and tested within existing systems of care to minimise information loss and risk to patients.

## Background

Handover is recognised as a high-risk process frequently associated with adverse events [1, 2]. It has been defined as the “*transfer of professional responsibility and accountability for some or all aspects of care for a patient, or groups of patients, to another person or professional group on a temporary or permanent basis*” [3]. Poor communication during patient handover has been identified internationally as a root cause of a significant proportion of preventable deaths [4]. Although the practice of handover between Ambulance and Emergency Department clinicians has received some attention over recent years [2, 5–7] there is little evidence on handover best practice within the pre-hospital domain. Pre-hospital services have therefore taken a pragmatic approach and developed their own systems and mnemonics to aide patient handover [2]. But few, if any of these, have been validated within the pre-hospital domain. There are other challenges. The abundance of available mnemonic’s [7], absence of agreed protocol and professional discretion may also be contributing to what could be described as a mnemonics confusion across systems. This is of particular concern as professional, social, environmental and human factors beyond the structured handover process have all been suggested as factors that influence handover effectiveness [2]. Many of these factors are likely to be amplified in the pre-hospital setting where there are multi-agency responses and clinicians manage patients in exposed, noisy, potentially dangerous environments with limited resource and clinical capabilities [6, 8–10]. It is unsurprising therefore, that handover has been highlighted as a WHO priority area for research [2, 11].

As trauma networks and specialist prehospital trauma teams have been developed to provide advanced medical and trauma care for time critical, high acuity patients [12–16] the importance of prehospital handover is increasingly apparent. Despite these teams being a very welcome addition to a prehospital care system, timely and accurate tasking of their resources remains a challenge [17]. Specialist prehospital teams often arrive as a secondary resource, thus necessitating clinical handover from road based ambulance clinicians. Although mnemonics do exist to support clinical handover these can lack content specificity and may be problematic in health care systems using different mnemonics [6, 18]. Furthermore, a recent review found little evidence to support the standardisation of handover processes and suggested that the function of mnemonics was uncertain [2]. Despite mnemonics being recommended for use between Ambulance Clinicians and Emergency Departments [19, 20], the suitability of these in the prehospital setting remains unknown and their use variable [21]. Further research is therefore urgently required to understand the most appropriate way to deliver clinical information exchange in the pre-hospital environment.

## Aim

To investigate current clinical information exchange practices, perceived challenges and the preferred handover mnemonic for use during transfer of high acuity, time critical patients between road based ambulance clinicians (RBAC) and specialist pre-hospital teams (SPHT).

## Methods

### Design

An online cross-sectional questionnaire.

### Setting

The study was undertaken in Scotland where the Scottish Ambulance Service (SAS) is the national provider of prehospital emergency care, covering 30,420 sqm, serving a population of 5.4 million [22] and responding to circa 560,000 emergency calls per annum [23]. The service is primarily set within an Anglo-American model of care [24] whereby road based Paramedics and Emergency Medical Technicians (EMT) deliver the majority of care. However, occasionally support is required from specialist paramedic teams with expertise on chemical, biological, radiological, nuclear (CBRN) and technical rescue, Helicopter Emergency Services (HEMS)/Search and Rescue (SAR), voluntary organisations such as British Association of Immediate Care (BASICS) and Physician led Specialist Prehospital Teams. Such extended services, particularly the inclusion of specialist critical care teams, provide an approach more akin to the Franco-German, physician led model of care [24].

### Pre-hospital specialist teams

SPHT are despatched through a variety of channels; i) on request by RBAC, ii) automatically via Medical Priority Despatch Systems, or iii) after clinical interrogation within the Ambulance Control Centre [25]. They offer unique knowledge, skills, equipment and resource not held by standard RBAC and respond to incidents across Scotland. For example, Special Operations Response Teams consist of Paramedics and Technicians (and non-clinical staff) who bring specialist knowledge, equipment, vehicles and resource to support operations in specific hazardous environments such as CBRN, water rescue and multi-casualty incidents. SAR, also paramedic led, provide care on behalf of the Maritime and Coastguard Agency, responding frequently to incidents in mountainous or coastal areas across Scotland (and the UK). HEMS provide a blended approach delivering paramedic led or Prehospital Critical Care Team led (Physician and Critical Care Practitioner) care tailored to the acuity level and clinical requirements of the patient/s. They provide critical care that includes advanced clinical decision making, induction and maintenance of anaesthesia, cardiovascular management and complex invasive interventions – of which most are currently beyond the scope of the UK based paramedic. BASICS responders, principally General Practitioners, provide a life-line of additional clinical support to RBAC in more remote and rural areas of Scotland where ambulance resources are scarce.

### Questionnaire development

A pragmatic three-stage approach was used to develop the questionnaire. Stage one: two authors undertook a scoping review of the literature to identify key papers on prehospital and emergency department handover. Key themes were identified, in particular barriers and facilitators, that along with clinical experiences of investigators, were used to inform the development of a draft questionnaire. These were formed into multi-choice questions or statements aimed at measuring the extent to which these factors impacted on prehospital handover. Key questions and areas of investigation, with their respective measures, are presented in Table 1:

**Table 1:** Key areas of questioning with scales/unit of measurement

Question/statement	Scale/Unit of measurement
• Perceived effectiveness of handover	1 – not at all effective to 5 - very effective
• Confidence that you have provided all essential information during handover	1 – not at all confident to 5 – very confident
• Confidence that you have received all essential information during handover	
• Importance of patient involvement in handover process	1- not important to 5 – very important
• Importance of a structured handover	
• Importance on mutually agreeing a handover time and location	
• Perceived essential variables for handover	List of variables
• Recording and delivery of information	
• Preferred mnemonic for prehospital handover	
• How professional acknowledges receipt of information	
• Acknowledging receipt of information	1 – never to 5 – always
• How often the patient is involved in the handover process	
• Barriers to effective handover	
• Repeating information during handover	
• Barriers to effective handover (how often they impact)	
• Difficulty in finding time to prepare and deliver handover	1 – very difficult to 5 – very easy
• Timing of handover	Time in minutes

Stage two: one key stakeholder from each of the specialist services (outlined below) reviewed and recommended changes to the questionnaire. These individuals were identified through the Scottish Ambulance Service professional networks and selected due to their role within their respective specialist team and expert knowledge in prehospital emergency care.

- SAS ambulance clinicians (road ambulance crew) – Paramedic and Technician led.
- Emergency Medical Retrieval Service/Tayside Trauma Team/Lothian Medic One clinicians – Physician led.
- Bristows Search and Rescue Aircrew – paramedic led.
- SAS Helimed aircrew (Inverness, Glasgow and Perth) – paramedic led.
- BASICS Scotland responders – mix of Physician, Paramedic and Nurse led.
- SAS Special Operations Response Teams Ambulance Clinicians – Paramedic and Technician led.

Stage three: an iterative process of further revisions by all authors produced a final draft questionnaire. This was sent to identified members of each service to test the questionnaire’s ease of comprehension and completion and led to a small number of revisions.

### Data definitions

A number of current handover mnemonics were included with participants’ afforded the opportunity to include additional mnemonics if theirs was not listed (Table 2).

**Table 2:** List of included mnemonics

Mnemonic	Breakdown
ASHICE	Age, Sex, History, Injuries, Condition, Expected Time of Arrival
DeMIST	DeMIST – Patient Demographics, Injuries Sustained, Symptoms and Signs, Treatments given
MIST	Mechanism of Injury, Injuries Sustained or suspected, Signs – vital signs, Treatments initiated (and timing)
SBAR	Situation, Background, Assessment, Recommendations
IMIST AMBO	Identification, Mechanism/Medical complaint, Injuries/Relevant info, Signs (vital), Treatment and Trends, Allergies, Medication, Background History, Other info
ATMIST	Age [inc. name], Time of onset, Medical Complaint/History or Mechanism, Investigations/Injuries, Signs, Treatment
De MIST	Patient Demographics, Mechanism, Injuries sustained or expected, Signs – vital signs, Treatment
SOAP	Subjective information, Objective Information, Assessment, Pain

### Study sample and recruitment

Existing ambulance data systems permit the identification of ambulance crews by call sign and then individual crew members by pay number. However, Ambulance Clinicians do not consistently and routinely record details that identify the crew member/s who provide or receive a handover. To ensure our questionnaire was targeted at the population under investigation a search of the ambulance call database was undertaken. This facilitated the identification of only those ambulances (call signs and therefore crew members) that had been in attendance at an incident involving any one of the pre-identified SPHT between July and September 2016 (the previous 3 months). Personalised invitations, informed by evidence-based methods aimed at improving response rates [26], were e-mailed to all clinicians who were involved in the identified incidents. To identify the members of the SPHT an e-mail invitation was also sent to a central co-ordinator in each of the SPHT who forwarded to the members of their respective specialist services, involved in the incidents identified, for completion. E-mails included a study information document and hyperlink to the online survey platform with supporting information video. Consent was presumed by completion of the questionnaire. A reminder e-mail was sent out after 3 weeks.

### Data analysis

Questionnaire results were analysed using SPSS v19. Summary statistics were presented as a frequency, percentage, median (M) and an interquartile range (IQR). Where relevant comparisons were made between RBAC and SPHT. Non-parametric measures were used to analyse these data, as their distribution was not normal, with a *p* < 0.05 deemed significant.

## Results

There were 247 pre-hospital incidents involving specialist teams over the 3 month study period. One hundred ninety individuals completed the questionnaire. Overall, responders were experienced prehospital care providers; number of years practicing Median 10 (IQR 5–18). Road-based ambulance clinicians made up 61% (*n* = 116) of participants with the remaining 39% (*n* = 73) from the five specialist services.

### Used and preferred mnemonics

**Fig. 1:**
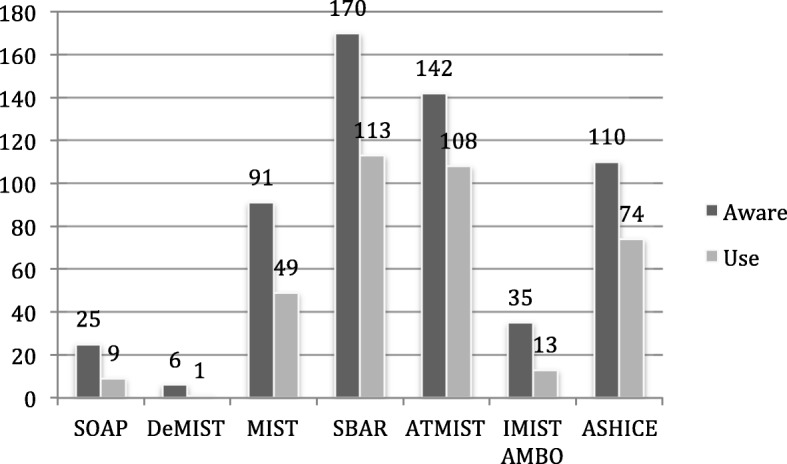
mnemonic awareness and usage across participants

All participants reported using more than one mnemonic. The three mnemonics with the highest frequency counts for both awareness and usage were SBAR, ATMIST and ASCHICE respectively (Fig. 1).

**Fig. 2:**
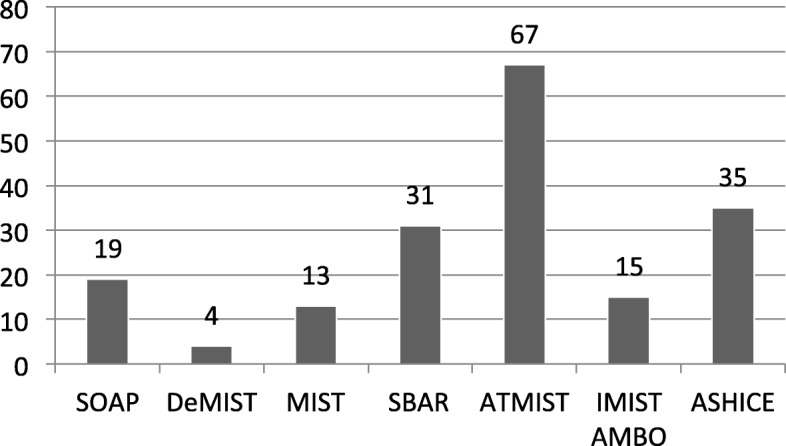
Mnemonic preference

The single preferred mnemonic for prehospital use was ATMIST; *n* = 67/184 (36%), followed by ASHICE *n* = 35/184 (19%) and SBAR *n* = 31/184 (17%) (Fig. 2).

### Perceived effectiveness and confidence in existing handover practices

Overall, two thirds of participants (68%; *n* = 130) reported handover as being either ‘*effective*’ or ‘*very effective’* (Mdn 4.00;IQR 3–4). Occasionally RBAC receive handovers, particularly where a SPHT has arrived before the ambulance resource. This is most likely to occur with HEMS where there is no requirement for aeromedical evacuation or where specialised teams have extricated a patient who subsequently required ambulance conveyance to the Emergency Department. It was therefore imperative to measure and compare both perspectives. SPHT reported a slightly lower perceived handover effectiveness rating than RBAC with 54% (*n* = 40) of SPHT compared to 78% (*n* = 90) of RBAC’s rating handover as either ‘*effective*’ or ‘*very effective*’; SPHT (Mdn = 4; IQR = 3–4) vs. RBAC (Mdn = 4; IQR = 4–4), *U* = 3344.0, *p* = 0.003.

When rating their personal confidence in the *provision* of essential information during handover, overall, participants scored a median rating of 4 (IQR 4–4) with 75% (*n* = 144) reporting feeling either ‘*confident*’ or ‘*very confident*’. There was no difference between RBAC and SPHT’s in self-reported handover confidence ratings. Conversely however, participants reported feeling less confident that they *received* all essential information during handover (*Mdn* = 3; IQR 3–4). Between-group analysis identified that those clinicians in the SPHT’s felt slightly less confident that they had received all essential information during handover (Mdn = 3; *IQR*2–4) when compared to RBAC (*Mdn* = 3; *IQR* 3–4), *U* = 3559.5, *p* = 0.03.

### Perceived essential variables for handover

**Fig. 3:**
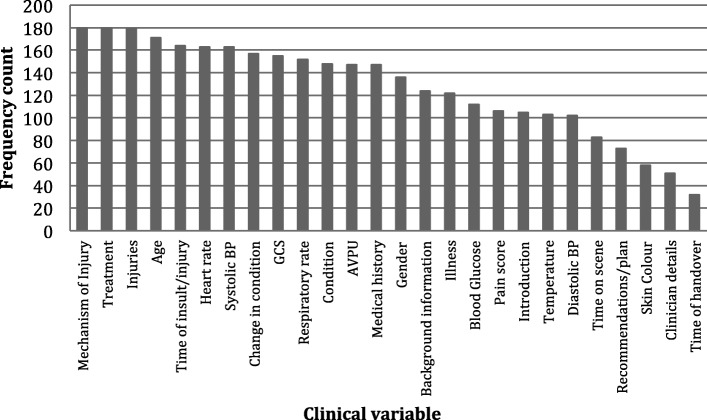
Frequency count of items felt essential for prehospital handover

Twenty-six variables were identified from published handover mnemonics. Participants were asked to select which of these they considered essential for delivery during handover. Figure 3 presents the frequency counts of participant’s responses. The participant’s prioritisation of essential clinical variables has high face validity with many of the higher priority variables independently, or in aggregation, being those that may provide an immediate clinical impression of the patient [27, 28]. Despite this, one particular variable stood out as receiving an unexpectedly low count; ‘illness’. This was noteworthy, as in practice the presenting condition is broadly categorised as either medical (illness) or trauma (injury). Although reasons for these differences were not explored, there are a number of possible explanations. First, our separation and ordering of variables within the questionnaire may have influenced participant’s selection. Second, many of the existing handover mnemonics omit the variable ‘illness’ [7] and so it is possible that this impacts clinicians’ awareness. And finally, it may be that the nature of calls requiring SPHT involvement (predominantly trauma) have influenced participant’s perceptions of the importance of specific variables.

### Barriers to effective handover

The occurrence of perceived potential barriers to handover were measured. A Likert scale was used to measure frequency (Never - 1 to Always – 5). Overall, ‘*interruptions’* received the highest mean rating, followed by ‘*Variability in handover mnemonic*’, ‘*Lack of co-ordination between responders*’ and ‘*Lack of structured process*’. Noteworthy too were the perceived frequency of ‘*lack of clear professional lead*’, ‘*poor verbal communication*’ and ‘*absence of written clinical information*’; all key components required to support a robust handover. Small but significant differences were found between the two groups mean rankings for three of the variables tested, ‘*lack of structured process*’, ‘*variability in handover*’ and ‘*environmental hazards*’. The specialist teams had a narrower distribution and more skewed towards ‘*sometimes*’ to ‘*often*’ (i.e. ratings 3 to 4 on the Likert scale of 1 – never to 5 – always) for both structured process and variability in handover (Table 3).

**Table 3:** Perceived barriers to prehospital handover

Variable measured (listed in order of frequency)	All Mean (Standard Deviation)	Road Crews (Standard Deviation)	Specialist Teams	All Median (IQR)	Road Crews (n = 116) Median (IQR)	Specialist Teams Median (IQR)	Difference between Groups *p* value (*U*)
Interruptions	3.26 (.813)	3.21 (.818)	3.35 (.801)	3.00 (2–3)	3 (2–3)	3 (3–4)	.224
Variability in handover mnemonic	3.09 (.953)	2.97 (.950)	3.28 (.929)	3.00 (2–4)	3 (2–4)	3 (3–4)	.034*
Lack of co-ordination between responders	3.09 (.761)	3.04 (.773)	3.16 (.741)	3.00 (3–4)	3 (3–4)	3 (3–4)	.222
Lack of structured process	3.07 (.879)	2.95 (.863)	3.26 (.877)	3.00 (2–4)	3 (2–4)	3 (3–4)	.022*
Lack of clear professional lead	3.01 (.813)	2.94 (.816)	3.11 (.804)	3.00 (2–4)	3 (2–3)	3 (3–4)	.141
Poor verbal communication	2.97 (.856)	2.90 (.882)	3.08 (.807)	3.00 (2–3)	3 (2–3)	3 (3–4)	.090
Absence of written clinical information	2.96 (.844)	2.91 (.875)	3.05 (.792)	3.00 (2–4)	3 (2–3)	3 (2.75–4)	.228
Hazards relating to the TYPE of incident	2.75 (.860)	2.66 (.814)	2.89 (.915)	3.00 (2–4)	3 (2–3)	3 (2–4)	.064
Environmental hazards	2.74 (.791)	2.62 (.798)	2.93 (.746)	3.00 (2–3)	3 (2–3)	3 (2–3)	.004*
Multi-agency involvement: too many	2.74 (.853)	2.71 (.856)	2.77 (.853)	3.00 (2–3)	3 (2–3)	3 (2–3)	.943
Difficulties in triage priorities during multi-casualty incident	2.67 (.795)	2.59 (.807)	2.79 (.763)	3.00 (2–3)	3 (2–3)	3 (2–3)	.204
Inappropriate location of handover	2.54 (.784)	2.46 (.832)	2.67 (.853)	3.00 (2–3)	3 (2–3)	3 (2–3)	.106
Lack of professionalism	2.54 (.872)	2.58 (.886)	2.49 (.852)	2.00 (2–3)	2 (2–3)	2 (2–3)	.673
Handover timing too early	2.48 (.762)	2.43 (.829)	2.54 (.645)	2.00 (2–3)	2 (2–3)	3 (2–3)	.354
Handover timing too late	2.40 (.783)	2.33 (.814)	2.50 (.726)	2.00 (2–3)	2 (2–3)	2.5 (2–3)	.117

*p* value obtained with Mann-Whitney U test; * donates a significant difference between RBAC and SPHT

### Views and experiences of the handover process

#### Preparatory effort, timing and location of handover

Although there were a high number of neutral responses (*n* = 77, 41% [Mdn = 3; IQR 2–3]), almost half of all responders (48%; *n* = 92) felt it was either ‘*difficult*’ to ‘*very difficult’* to find time to prepare for prehospital handover.

Perceptions of time (in minutes) required for handover preparation and then delivery were measured separately in minutes and show a bi-modal distribution. For preparation, overall, 77.4% (*n* = 147) of participants felt that up to 3 min was required. However, almost 20% (*n* = 37) of responders felt they required up to 5 min to prepare. On the time required to deliver a handover, 91% (*n* = 174) of participants stated they required up to 3 min. From this sub-group of participants, the largest proportion (57%; *n* = 100/174) reported they required only up to 1 min to deliver a handover. The need to identify an appropriate ‘*location*’ and ‘*time*’ for handover was felt to be either ‘*important*’ to ‘*very important*’ in 77% (*n* = 145) and 69% (*n* = 131) of participants respectively; (‘*Location’* Mdn = 2; IQR 1–2; ‘*Timing’* Mdn = 2; IQR 1–2).

#### Acknowledging receipt of information

Participants’ experiences of three aspects of post-handover feedback were sought. 41% (*n* = 78) of participants reported they ‘*often*’ or ‘*always*’ received immediate acknowledgement of their handover, with 50% (*n* = 94) only ‘*sometimes’*. When feedback was received this was mostly via a verbal ‘*thank you*’ (81%; *n* = 153), however 51% (*n* = 96) did also report those receiving handover perform a ‘read back’ of the information provided. 84% (*n* = 158) of participants stated they ‘*sometimes’*, ‘*often’* or ‘*always’* had to repeat information during handover.

#### Recording and delivery of information

All SAS ambulance clinicians are issued with paper-based clinical guideline pocket books (size A6) that contain handover mnemonics within. However, these do not contain corresponding field boxes to facilitate clinical data recording. Our questions therefore focused on the participant’s method/s to record and deliver clinical data for handover, rather than on whether they used any particular mnemonic card as an ‘aide memoire’ to support this process. To ensure a more accurate representation of current practices, and identify possible inconsistencies in clinical data recording and delivery, participants were permitted to select from a range of possible supporting recording and delivery methods. As such, unsurprisingly, there was considerable variation in participant’s responses. The most frequently reported methods for the recording and delivery of clinical information during handover were ‘*committed to memory’* (60%; *n* = 113) and ‘*verbally from memory’* (63%; *n* = 120) respectively. Also commonly used were electronic Patient Report Forms (ePRF) and scrap paper to record and support the delivery of clinical information used to support handover; ePRF recording (60%; *n* = 112) and delivery (57%; *n* = 109); scrap paper for recording (51%; *n* = 97) and delivery (37%; *n* = 71).

#### Involving patients in the handover process

58% of participants (*n* = 110) felt it was either ‘*important*’ or ‘*very important*’ to involve the patient in the handover process, with 27% (*n* = 52) expressing neutral thoughts and the remaining 15% (*n* = 28) ‘*unimportant*’ or ‘*not at all important*’. There was a positive correlation between those who felt it important to involve patients in handover and self-reported involvement of patient during handover (*r* = .617, *n* = 190, *p* < .001).

## Discussion

This study, to our knowledge, is the first to investigate handover between prehospital road based ambulance clinicians and specialist prehospital services. One hundred ninety experienced prehospital practitioners, who were involved in 247 incidents over a four month period, completed the questionnaire. While handover was generally thought to be effective, practices appear highly variable across Scotland. The recommended SBAR mnemonic is not always used, nor is it the preferred mnemonic for supporting prehospital handover. Furthermore, this study has established that there are diverse challenges that impact on the exchange of clinical information between those primarily providing (RBAC) and receiving (SPHT) essential clinical information. Barriers to effective handover were apparent and occasional small differences in opinions on handover quality were identified between the SPHT and the RBAC. Collectively, these exposed challenges are of concern, but this new understanding provides us with an opportunity to focus on service improvement and further research.

### Key challenges

The gathering, synthesis, construction and delivery of a detailed yet succinct handover demands considerable cognitive effort as well as time. Our study confirms that the handover challenges identified within the prehospital setting are similar to those identified within the ED literature [29] and thus some of the previously developed evidence may be transferrable to the prehospital setting. There were a number of elements identified within the current system that appear to impede the handover process and that require attention.

First, many participants reported it difficult to generate time to prepare for handover, a particular challenge within a resource-limited setting. Preparation is often inadequate but forms an essential component of the handover process [11]. The current adhoc approach during the preparation and exchange of clinical information will likely add to an already increasing cognitive burden. Indeed Cognitive Load Theory has recently been used to further understanding of the complexities of handover and has determined that multiple factors are associated with different types of cognitive load; sensory, working and long term memory [30]. The ‘working memory’ used during handover is finite, with limited capacity, being capable of holding only 4 to 7 (± 2) ‘units’ of information at a time [30]. It is reasonable therefore to suggest that the current variable preparatory process is detracting clinicians either in the preparation of a handover or from other important aspects of care delivery. These factors may also impact negatively on patient safety [31–33].

Second, our study exposed the relatively frequent absence of an identified professional lead as a barrier to handover. Participants also highlighted the importance of agreeing the timing and location of handover. It would be difficult to undertake the latter in the absence of a clear ‘lead’ and indeed other studies have demonstrated significant improvements in clinical care from the introduction of an active team lead [34, 35]. Where a clear professional lead is identified, the initial engagement required to establish the timing and location of handover may enable the restoration of a degree of control in these challenging environments and is therefore recommended.

Third, although we did not fully explore the physical format in which participants used aide-memoires to support handover, we did investigate participants’ methods of clinical data recording and subsequent transfer during handover. The preference by many to use ‘*memory’* to both record and deliver handover information is concerning. Previous research has identified that only 33% of data is retained on first handover when relying on memory alone, but where standardised, printed forms are used, data retention can increase to almost 100% [36]. One study within the ED setting demonstrated less than 50% of the information provided during paramedic handover was retained by ED staff [37]. As has previously been discussed, cognitive load will be high during such incidents. It is reasonable to presume then, when key clinical information is not written down or recorded, some will be forgotten or imprecise recall will interfere with the sharing of accurate clinical data. Studies from the in-hospital environment have reported similar issues in the delivery and receipt of handover information [5, 6]. Undoubtedly, these issues too have the potential to impact on patient safety. Although our data indicates many participants also reported using the electronic Patient Report Form (ePRF) to record information for handover, during high acuity calls the ePRF often remains in the treatment area of the ambulance. This technology is therefore not immediately available to the RBAC. The ePRF is used, but often completed retrospectively, evidence also by the recognised frequency of an ‘*absence of written clinical information’* as a barrier to effective handover (Table 3). These clear limitations to the existing ePRF system result in RBAC, during the incident, often resorting to the manual, contemporaneous recording of these data on scrap paper or the back of a gloved hand. A practice also identified by others [38, 39] and one that is not without its own inherent risks.

And fourth, the inconsistent use of mnemonics, lack of co-ordination and structured processes were all identified as key issues in handover. Manser and Foster [1] recommend the development of a share mental model for handover between teams, however this is unachievable where such inconsistencies exists. The importance of consistency in handover is also emphasised in the study by Starmer et al. [40] where a 23% decrease in medical error rate post-introduction of a standardised handover process was reported. The issues on consistency in our study may have therefore contributed to ‘*interruptions’* being identified as the most frequent barrier to effective handover. Interruptions can occur during handover when the receiver seeks information pertinent to them that was missed by the provider. It is recognised from in-hospital handover literature that the perceived quality of handover is dependent on the expectations of those receiving it [5]. This may also provide some explanation as to why SPHT rated handover quality slightly lower than RBAC – expectations of handover differed. Similar difficulties, particularly around interruptions, have also been described within the Emergency Department setting [5, 9, 41]. Notably these have been identified as a potential contributor to clinical error [41]. Such expectations may be managed more effectively, therefore, via the introduction of a shared mental model of handover [11, 42]. These must be considered within the context of any future handover process introduced.

### Considerations on developing more effective handover

Providing high quality handover is dependent on multiple factors. To determine that there exists national inconsistencies in handover approach was of significant concern. And in recognising that there is currently no ideal, evidence-based and definitive solution to this challenge, clinicians should reflect on the existing evidence to determine whether the introduction of a nationally agreed and standardised mnemonic could support prehospital handover by reducing variability and, therefore, the recognised mnemonics confusion. It would be illogical to ignore the evidence that is available, particularly that which suggests that an agreed, standardised mnemonic can reduce handover duration, repetition, improve structure and consistency and also the promotion of the shared mental model concept [1, 6].

Solutions that are more pragmatic may also lend support to reducing cognitive demand during handover. For example, the reliance on memory to record and deliver clinical information may indicate a need to develop some novel interventions to ease these processes for prehospital clinicians. There are known low [43] and high tech solutions [38, 39] available that possess the potential to free up significant time and therefore cognitive effort required for handover preparation and delivery in these high fidelity settings. The need to identify a clear handover lead, as has been previously discussed, is also essential and should be incorporated into any system. Although, developing these skills would rely on additional education and rehearsal in handover [5] and increased resource. Identification and understanding these many factors that impact on handover emphasise the complexities of handover and the predictable need for a multi-modal intervention to support the process. And so, in the absence of high quality evidence there remains a need for greater understanding in this area. New theoretically informed [1], evidence-based interventions, must be developed and tested within existing systems of care.

### Limitations

#### Internal validity

The true efficacy of handover would require more objective, validated measures [1]. We invited all individuals who attended these incidents over the study period to participate, however, there may have been response bias in those that chose to respond to the invitation and participate. It was also likely that some relevant questions were not included but we attempted to minimise this through an iterative approach to questionnaire design and the inclusion of experts in prehospital care in its development.

#### External validity

This study provides an understanding of the perceptions and experiences of key professionals providing prehospital care across Scotland. The transferability of these results to prehospital services beyond Scotland, and the UK, is not known, but should be acknowledged as a limitation of this study. We did not obtain the views of certain groups such as Mountain Rescue, Community First Responders, Fire and Rescue as we decided to include only those registered as Medical, Nursing, Health Care Professionals and EMT’s. As with all surveys a self-selection bias may impact on the results of this study.

## Conclusion

No previously published study has investigated the practice of prehospital handover between RBAC and SPHT. Despite the overall positive perceptions of handover our study identified significant practice and mnemonic variation across Scotland. These variations were apparent at individual level, between participants and between prehospital teams. Although Wood et al. [2] concluded that mnemonics alone do not necessarily improve handover, there is some evidence to suggest they can reduce variability [6] and as part of a broader handover system can significantly reduce medical errors [40]. Our results were therefore of concern. However, we now have evidence of the practical challenges that prehospital teams face during handover; barriers that affect handover, concerns around contemporaneous data recording and the need to have a clearly identifiable handover lead. This knowledge could support future improvements in handover. This study provides a comparator (or benchmark) for future investigations in this area of care.

The results and associated concerns have also been discussed within the context of the available literature. Perhaps unsurprisingly exposing that prehospital and in-hospital handover share similar challenges. There is little published evidence of the risks associated with prehospital handover, however medical error rates associated with handover in hospital are well evidenced and are a significant problem [3, 40]. Given these similarities in handover challenges, prehospital providers would be unwise to ignore this risk due to a lack of published evidence within their own professional domain. These similarities should motivate us to question whether the demonstrable success in improving handover within hospital, in particular reducing medical error rates [40], are reproducible within the prehospital setting. This conclusion strengthens the need for research in this area of care. Further objective measures of handover quality (subjective and objective) are required, including medical error rates, on which the success or failure of future interventions may be measured. Although the pragmatic mnemonic alone may lack the power to provide a definitive solution to the handover problem, there is merit in including this as a part of a theoretically informed, multi-modal intervention within the context of the shared mental model [11].
